# Teleoncology or telemedicine for oncology patients during the COVID-19 pandemic: the new normal for breast cancer survivors?

**DOI:** 10.2217/fon-2020-0714

**Published:** 2020-08-28

**Authors:** Fatih Yildiz, Berna Oksuzoglu

**Affiliations:** ^1^Department of Medical Oncology, University of Health Sciences, Dr AY Ankara Oncology Training & Research Hospital, Ankara 06200, Turkey

**Keywords:** breast cancer, COVID-19, telemedicine, teleoncology

## Abstract

**Background:** Telemedicine is seen as a savior during the COVID-19 pandemic. **Materials & methods:** This study is a descriptive cross-sectional study conducted with cancer patients who were interviewed via telemedicine from a tertiary care comprehensive oncology center. **Results:** A total of 421 patients were included in the study and 118 of them (28.0%) were >65 years old. Communication was provided most frequently by voice call (n = 213; 50.5%). The majority of the patients contacted by telemedicine had breast cancer (n = 270; 64.1%). For 135 patients (32.1%) no further examination or intervention was required and the previously planned follow-up visit was postponed by the clinician. **Conclusion:** This study showed that telemedicine could open a new era for medical oncology specialists.

The Coronavirus disease-19 (COVID-19) pandemic, which started in December 2019 in Wuhan, China, had spread over the world by June 2020 [1], including Turkey. As of June 1, 2020 6,339,060 people around the world and 164,769 people in Turkey had a COVID-19 diagnosis [1]. We have learned from the Chinese and European experiences that mortality is higher in COVID-19 in patients with chronic diseases, immune suppressive conditions and advanced age [2,3]. It is obvious that oncology patients are at higher risk for COVID-19 complications [4].

This situation, which we have encountered for the first time, directed oncologists to take some precautions in order to protect their patients. European and American guidelines recommend, among other things, the use of oral treatments where possible, considering chemotherapy holidays for patients receiving palliative treatment, and postponing controls of oncology survivors. The use of telemedicine is another of these suggestions [5,6].

Recent dizzying advances in technology have induced important changes in the medical field, as in other fields. The increase in internet and smartphone usage in recent years has made communication easier and faster. It is possible to advise, intervene and direct patients by providing remote access via telemedicine or telehealth, which is defined as video, audio or written communication with patients [7,8]. Telemedicine, which has previously been underutilized, is seen as a savior during the COVID-19 pandemic [9,10]. On 13 March, 2020, with the emergency act stated in USA, telemedicine was officially allowed and reimbursed [9]. The applicability of the telemedicine concept – which most centers, including ours, experienced for the first time in the COVID-19 process, and other centers started to use more frequently – is discussed in relation to future periods after the pandemic [11].

Is telemedicine an effective method for oncology patients? In other words, can telemedicine meet the demands of oncology patients and is it an effective method for solving problems? During the days of the ‘stay at home’ warning, how many of the oncology survivors’ demands were met by the use of telemedicine, without the need of a visit to the hospital? During the pandemic period, did patients living in different provinces avoid risky trips with the help of the telemedicine method? The aim of this study is to share the experience we have gained through the implementation of telemedicine methods during the COVID-19 pandemic.

## Materials & methods

Our hospital is a tertiary care comprehensive oncology center which admits an average of 400 solid malignancy patients per day to its medical oncology outpatient clinics. Approximately one-third of these patients are cancer survivors and patients on oral therapy.

This study is a descriptive cross-sectional study which was conducted between April 22 and June 1, 2020 with oncology patients who were interviewed via the telemedicine communication line of our hospital's medical oncology department. The telemedicine communication line was activated on April 22 in our clinic. In this process, communication with patients was carried out by a single medical oncology specialist. All patients contacted for telemedicine were evaluated for inclusion to the study. Patients with solid malignancies receiving oral treatment in metastatic or nonmetastatic stages and cancer survivors who had completed their treatments were included in the study. Patients receiving intravenous systemic therapy (chemotherapy, immunotherapy, monoclonal antibody) were excluded because they were directed to the ‘chemotherapy hotline’. Data of all patients meeting inclusion criteria were evaluated in the study.

Demographic characteristics, diagnosis and stage of the disease, oncological treatment in progress, communication method (video calling, WhatsApp or short messaging service, voice call) and decisions taken during the interview on treatment or follow-up were recorded. Administrative approval was obtained from the Ministry of Health and ethical approval was obtained from the local board of ethics prior to the study.

Descriptive analysis was performed with the SPSS V.21 statistics program.

The primary end point of the study was to determine the proportion of patients using telemedicine whose demands were met without the need for in-person care. The secondary end points were to measure the proportion of patients who did not have to apply to any healthcare center and the proportion of patients living in different provinces who did not have to come to our center because they used telemedicine.

## Results

A total of 421 patients were included in the study and 342 (81.2%) of them were women. Median age was 55 years (range: 25–82) and 118 patients (28.0%) were >65 years old. Most of the patients (n = 277; 65.8%) were living in the capital city where our hospital is located; however, 141 patients (33.5%) were resident in different provinces and 3 patients (0.7%) were living in different countries.

The majority of the patients contacted by telemedicine had breast cancer (n = 270; 64.1%). A further 48 (11.4%) patients had colorectal cancer and 21 (5.0%) had gynecological cancer. Of all patients, 130 (30.9%) had metastatic cancer, 253 (60.1%) were receiving hormone therapy and 48 (11.4%) were receiving oral chemotherapy or oral targeted therapy. About 28.5% (n = 120) of the patients were not receiving any treatment. Patient characteristics are shown in Table 1.

**Table 1.** Patient characteristics.

Characteritics	n = 421	%
**Age**		
Median (range), years	55 (25–82)	
**Sex**		
Male	79	18.8
Female	342	81.2
**Type of malignancy**		
Breast	270	64.1
Colorectal	48	11.4
Gynecological	21	5.0
Prostate	13	3.1
Lung	12	2.9
Others	57	13.5
**Clinical tumor stage**		
Nonmetastatic	291	69.1
Metastatic	130	30.9
**Type of treatment**		
Hormone therapy	253	60.1
Oral chemotherapy	28	6.6
Oral targeted therapy	20	4.7
None	120	28.5

Communication was most frequently provided by voice call (n = 213; 50.5%). Video interview was used for 42 (9.9%) patients and text message was used for 166 (39.4%) patients. Details of patients’ clinical features according to the method of communication are provided in Table 2.

**Table 2.** Clinical features of patients according to the method of communication.

Characteristic	Voice call (n = 213)	Video interview (n = 42)	Text message (n = 166)
**Type of malignancy**			
Breast	148 (54.8%)	15 (5.5%)	107 (39.6%)
Colorectal	21 (43.7%)	6 (12.5%)	21 (43.7%)
Gynecological	10 (47.6%)	2 (9.5%)	9 (42.8%)
Prostate	3 (23.0%)	3 (23.0%)	7 (53.8%)
Lung	5 (41.6%)	3 (25.0%)	4 (33.3%)
Others	26 (45.6%)	13 (22.8%)	18 (31.5%)
**Clinical tumor stage**			
Nonmetastatic	134 (46.0%)	12 (4.1%)	145 (49.8%)
Metastatic	79 (60.7%)	30 (23.0%)	21 (16.1%)
**Type of treatment**			
Hormone therapy	135 (53.3%)	12 (4.7%)	106 (41.8%)
Oral chemotherapy	8 (28.5%)	15 (53.5%)	5 (17.8%)
Oral targeted therapy	8 (40.0%)	10 (50.0%)	2 (10.0%)
None	62 (51.6%)	5 (4.1%)	53 (44.1%)

The demands of 92.8% (n = 391) of our patients using telemedicine were met without the need for in-person care and 93.0% of the 144 patients (n = 134) living in different provinces had their problems solved without having to travel to our center. For 135 patients (32.0%) no further examination or intervention was required and any previously planned follow-up visit was postponed by the clinician at the end of the interview. Of these patients, 109 (25.8%) had nonmetastatic breast cancer, 15 (3.5%) had nonmetastatic colorectal cancer and 4 (0.9%) had nonmetastatic gynecological cancer.

In order to perform laboratory tests or radiological imaging, 159 (37.7%) patients were referred to local healthcare centers in their residency area and the results were evaluated without any need to visit our hospital. 50 (11.8%) of these patients had metastatic breast cancer, 16 (3.8%) had nonmetastatic breast cancer, 10 (2.3%) had metastatic colorectal cancer and 9 (2.1%) had nonmetastatic gynecological cancer. Subcutaneous or intramuscular treatments (LHRH analogs, bisphosphonates, fulvestrant) were ordered electronically and injections were administered to 97 patients (23.0%) in our outpatient treatment unit without the need for waiting in the outpatient clinics registry. 70 (16.6%) of these patients had nonmetastatic breast cancer, 15 (3.5%) had metastatic breast cancer and 5 (1.1%) had metastatic prostate cancer. 30 (7.1%) patients were called to the medical oncology department. Half of these patients (n = 15, 3.5% of the total patients in the study) were invited to our clinic in order to perform a comprehensive physical examination and the other half required in-person care for urgent intervention.

## Discussion

The COVID-19 pandemic that has affected the whole world is considered to be a new milestone in many areas. During this period, health professionals are taking different measures to protect their own patient groups. Telemedicine is one of the methods we are experiencing as oncologists. In this study we aimed to share our first telemedicine experience with cancer patients during the COVID-19 pandemic. To our knowledge, this is one of the most comprehensive studies involving the telemedicine method in oncology patients during the COVID-19 pandemic.

One of the decisions taken by Turkey’s central government was the lockdown of people over 65 years old except in special circumstances, beginning from 21 March, 2020. In our study 28% of patients who benefited from telemedicine were among this population. With the use of telemedicine, healthcare services were provided via remote communication to the patients at high risk for morbidity and mortality due to cancer, comorbid diseases and advanced age.

In Turkey, another measure taken by the government was to ban entry to and exits from metropolitan areas, except in special circumstances. Our hospital was located in one of the cities within this scope. In this period, 33.5% of our patients who were contacted through telemedicine lived in different provinces. Telemedicine was a necessary approach and it proved very useful, because exhausting formal procedures were required for both oncology patients and their companions, and public transportation, intercity planes and buses were restricted.

According to the results of our study, the patients who derived the most benefit from telemedicine were those who had a breast cancer diagnosis; the majority of all patients included in the study were in the nonmetastatic stage. This situation enabled about 30% of patients interviewed via telemedicine to be evaluated without any examination or intervention and their follow-up visits were postponed. While it may be possible to evaluate an early stage breast cancer patient with minimal examination or remote communication, it would be very difficult in a newly diagnosed patient or one with metastatic cancer of unknown primary. Therefore it is crucial to select carefully the appropriate patient group that can benefit from telemedicine in terms of making minimal mistakes in the treatment and follow-up processes. One of the best examples of telemedicine experiences during the pandemic was performed by Kang *et al.* In this study, 27% of patients with head and neck cancer required in-person care for a status check, whereas in follow-up visits no in-person care was required [12].

In most of the previous reviews, telemedicine was defined as the evaluation of patients by a healthcare professional via a written, audio or video method [8,9]. We took it one step further, beyond evaluation, in the pandemic period. Short-term intravenous, intramuscular and subcutaneous treatments, such as fulvestrant, bisphosphonate and LHRH, were prescribed by e-order in the outpatient treatment unit for 97 patients (23.0%) who were evaluated by the medical oncology specialist with telemedicine. These patients avoided the waiting time required for in-person care in the medical oncology outpatient clinic in routine practice. The risk of COVID-19 contamination in crowded outpatient clinic waiting rooms has been reduced to minimum for these patients.

The limitations of this study were, firstly, that patients who had been receiving active systemic intravenous administration of chemotherapy, immunotherapy and monoclonal antibodies, and those who had a planned hospitalization, were excluded from the study because they were evaluated by a separate communication line. Another limitation was that the study has no data on patients’ perception of satisfaction. In most of the studies on telemedicine before the COVID-19 pandemic, patient satisfaction has been seen as one of the most important key points for success. A new study on this subject has been considered for the future. However, we can say that the telemedicine method has reached its intended goals. The clinical problem was solved and the demands were met for 92.9% of the patients without the need for an invitation to the center for in-person care.

One of the opportunities offered by the COVID-19 pandemic was that it enabled us to experience telemedicine with oncology patients. With this system it seems possible to minimize the risk of infection acquired from the hospital, both for patients and healthcare providers. Another advantage was that it allowed us to use less personal protective equipment. In this way it became possible to transfer this equipment to more critical units, such as intensive care units. There are also disadvantages of the system; for example, the lack of a full physical examination and the lack of in-person care, which may cause patient distrust. Other deficiencies are that the system does not have an authorized description in our country or in many other countries; therefore, the costs may not be covered or reimbursed by insurance systems.

## Conclusions

This study showed that telemedicine could open a new era for oncology specialists, especially for clinics heavily loaded with breast cancer patients. After the COVID-19 pandemic, further application of this method should be seriously discussed. Although it may have disadvantages, it should not be forgotten that in unusual situations such as pandemics, telemedicine or teleoncology may be a good alternative. Further studies would help us evaluate the efficiency of telemedicine by comparing telemedicine users with in-person care patients.

## Future perspective

In our opinion, telemedicine will be more widely used among oncology specialists in the coming years. With the widespread use of the internet in developing countries, patients would prefer video calling more than other methods as a communication option and may want to meet with clinicians more frequently. Perhaps telemedicine will be preferred more than in-person care by oncology survivors.

**Executive summary**

- The Coronavirus disease-19 (COVID-19) is more fatal in patients with chronic diseases such as malignancy.
- During this period, health professionals tried to take different measures to protect their own patient groups.
- Telemedicine is one of the methods that we had to utilize as oncologists.
- In this study we aimed to share our first experience of telemedicine with cancer patients during the COVID-19 pandemic.
- The patients who benefited from telemedicine were mostly those who were diagnosed with breast cancer; the majority of all patients included in the study were in the nonmetastatic stage.
- For 135 patients (32.0%), no further examination or intervention was required and previously planned follow-up visits were postponed by the clinician at the end of the interview.
- This study showed that telemedicine could open a new era for oncology specialists, especially for clinics heavily loaded with breast cancer patients.
- After the COVID-19 pandemic, further application of this method should be seriously discussed.

## References

[1] WHO. WHO Coronavirus Disease (COVID-19) Dashboard (2020). https://covid19.who.int/

[2] Xia Y, Jin R, Zhao J Risk of COVID-19 for patients with cancer. Lancet Oncol. 21(4), e180 (2020). 32142622 10.1016/S1470-2045(20)30150-9 PMC7130057

[3] Sorbello M, El-Boghdadly K, Di Giacinto I Società Italiana di Anestesia Analgesia Rianimazione e Terapia Intensiva (SIAARTI) Airway Research Group, and The European Airway Management Society. The Italian coronavirus disease 2019 outbreak: recommendations from clinical practice. Anaesthesia 75(6), 724–732 (2020). 32221973 10.1111/anae.15049

[4] Zhang L, Zhu F, Xie L Clinical characteristics of COVID-19-infected cancer patients: a retrospective case study in three hospitals within Wuhan, China. Ann. Oncol. 31(7), 894–901 (2020). 32224151 10.1016/j.annonc.2020.03.296 PMC7270947

[5] European Society for Medical Oncology (ESMO). Guidelines (2020). www.esmo.org/guidelines/covid-19-adapted-recommendations-slide-sets

[6] American Society of Clinical Oncology (ASCO). ASCO special report: guide to cancer care delivery during the COVID-19 pandemic (2020). www.asco.org/sites/new-www.asco.org/files/content-files/2020-ASCO-Guide-Cancer-COVID19.pdf

[7] Hau YS, Kim JK, Hur J How about actively using telemedicine during the COVID-19 pandemic? J. Med. Syst. 44(6), 108 (2020). 32350626 10.1007/s10916-020-01580-z PMC7190457

[8] Ghosh A, Gupta R, Misra A Telemedicine for diabetes care in India during COVID19 pandemic and national lockdown period: guidelines for physicians. Diabetes Metab. Syndr. 14(4), 273–276 (2020). 32283497 10.1016/j.dsx.2020.04.001 PMC7129346

[9] Doarn CR, Merrell RC The day the earth stood still: COVID-19. Telemed. J. E-Health 26(5), 569–570 (2020). 32384021 10.1089/tmj.2020.29039.crd

[10] Gill S, Hao D, Hirte H Impact of COVID-19 on Canadian medical oncologists and cancer care: Canadian Association of Medical Oncologists survey report. Curr. Oncol. 27(2), 71–74 (2020). 32489248 10.3747/co.27.6643 PMC7253737

[11] Bashshur R, Doarn CR, Frenk JM Telemedicine and the COVID-19 pandemic, lessons for the future. Telemed. J. E-Health 26(5), 571–573 (2020). 32275485 10.1089/tmj.2020.29040.rb

[12] Kang JJ, Wong RJ, Sherman EJ The 3 B's of cancer care amid the COVID-19 pandemic crisis: ‘Be safe, be smart, be kind’ – A multidisciplinary approach increasing the use of radiation and embracing telemedicine for head and neck cancer. Cancer 10, 1002 2020). 10.1002/cncr.33031 PMC7361524 32639615

